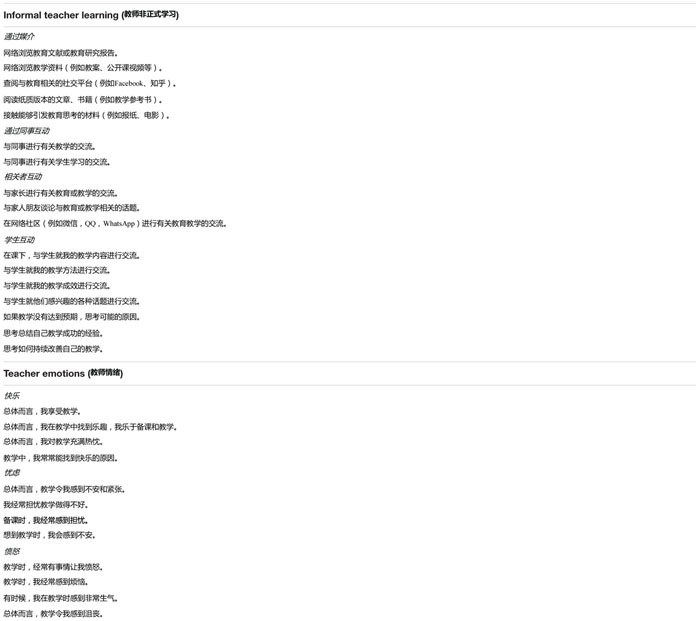# Striving to Become a Better Teacher: Linking Teacher Emotions With Informal Teacher Learning Across the Teaching Career

**DOI:** 10.3389/fpsyg.2020.01067

**Published:** 2020-05-27

**Authors:** Xianhan Huang, John Chi-Kin Lee, Anne Christiane Frenzel

**Affiliations:** ^1^Faculty of Education, The University of Hong Kong, Pokfulam, Hong Kong; ^2^Department of Curriculum and Instruction, The Education University of Hong Kong, Tai Po, Hong Kong; ^3^Department of Psychology, Ludwig Maximilian University of Munich, Munich, Germany

**Keywords:** teacher emotions, enjoyment, anxiety, anger, informal teacher learning, teaching experience, TES-Chinese

## Abstract

The importance of informal teacher learning (ITL) to teaching effectiveness and student achievement has been repeatedly demonstrated, but there is limited research into the personal antecedents of ITL. We analyzed the relationships between teacher emotions and participation in five different kinds of ITL activities (learning through media, colleague interaction, stakeholder interaction, student interaction, and individual reflection) among 2,880 primary teachers (85.49% female) with a large range of teaching experience. Regression analysis and structural equation modeling revealed a positive association between enjoyment and engagement in all five ITL activities. Anxiety was found to be negatively related to colleague interaction and self-reflection, and anger was found to be negatively associated with student interaction. Furthermore, anxiety and anger were negatively related to teaching experience, whereas enjoyment was independent from teaching experience. Most ITL activities were positively related to teaching experience, except for stakeholder interaction. Implications for teacher training and intervention programs for in-service teachers are discussed.

## Introduction

Teachers are expected to continue learning throughout their careers so they can manage uncertain and complex classroom situations and cater to changing societal needs (Day and Sachs, 2004; Loughran and Hamilton, 2016). Continued learning on the part of teachers is acknowledged as an important predictor of student learning (Darling-Hammond, 2008), teacher professional development (Day et al., 2007), and school improvement (Opfer and Pedder, 2011). As a core constituent of continued learning (OECD, 2003), teachers have reported that informal learning plays a decisive role in their professional development (Kyndt et al., 2016). With growing awareness of the potential of informal teacher learning (ITL), some studies have examined what activities contribute to ITL in various educational contexts and how different factors encourage or discourage engagement in these activities (Lohman and Woolf, 2001; Henze et al., 2009; Hoekstra et al., 2009; Bakkenes et al., 2010). A number of personal factors have been identified as related to ITL, including personality (Fox et al., 2011), will to learn (Van Eekelen et al., 2006) and self-efficacy (Runhaar et al., 2010; Van Daal et al., 2014). Although researchers have begun to examine the affordances of participation in ITL, most attention has focused on how personal cognition or motivation influences ITL; much less is known about how emotional traits promote engagement in ITL activities (Benozzo and Colley, 2012).

The inextricable link between emotion and cognition has captured the interest of educational scientists (Pekrun, 2006; Korthagen, 2017; Graesser, 2019) and has been demonstrated in different fields, such as psychology (Deci and Ryan, 2002; Pekrun, 2006; Immordino-Yang and Damasio, 2007; Tyng et al., 2017), multimedia learning (Knörzer et al., 2016), and artificial intelligence in education (Forbes-Riley and Litman, 2010; Calvo and D’Mello, 2011). In the specific field of ITL, however, it seems that no study to date has explored how teachers’ emotions are related to their engagement in ITL activities. Despite broad acknowledgment of the general link between affective and cognitive states in learning, the kinds of emotions that influence different kinds of ITL activities and how exactly these relationships are constituted remain unexplored. With its exploration of the relationship between teacher emotions and ITL activity, this study is among the first to discuss how emotions are linked with teachers’ learning behavior. We provide evidence for the significance of teacher emotions to teacher workplace learning by addressing the possible role that specific teacher emotions play in promoting or prohibiting ITL activities. Furthermore, the study extends the current understanding of the complex effects of teacher emotions through a detailed examination on how enjoyment, anxiety, and anger are related to specific types of learning activities. The findings can yield a clear picture of how various teacher emotions are integrated into teachers’ workplace learning activities, which has implications for policies regarding the continuous professional learning and emotional well-being of teachers.

It has been demonstrated that teachers’ beliefs and behaviors undergo significant changes over the course of their teaching careers (Day et al., 2007; Klassen and Chiu, 2011; Richter et al., 2011). However, little is known about how teachers’ emotions and ITL activities vary with teaching experience (Frenzel, 2014; Richter et al., 2011). Despite a fairly weak empirical basis, there are some indications that beginning teachers tend to be more anxious about teaching (Intrator, 2006) and to engage in more teaching collaborations (Richter et al., 2011), and that teachers in the late-career stage have lower learning intentions (Van Daal et al., 2014). However, there has been no systematic exploration of how particular emotions and engagement with different kinds of ITL vary with the range of teaching experience, despite the value that findings in this area would provide for the effective design of emotional interventions and learning improvement programs for different teachers. The results of this study can therefore provide policymakers and researchers with useful insights into ways to adapt related interventions and programs to the different needs of teachers with various levels of teaching experience.

To address the research gap, this study was designed to explore teacher emotions and ITL activities among teachers with a large range of teaching experience. First, we focused on mapping the relationships between three prominent emotions experienced by teachers (enjoyment, anxiety and anger), and five ITL activities. Furthermore, we examined how emotions and ITL activities varied with teaching experience. With this design, the findings can be of benefit not only to theoretical understandings of learning-related emotions but also to practices of in-service ITL.

### Informal Teacher Learning (ITL)

As a result of shifts in the workplace, with knowledge now being increasingly considered a crucial component of work (Ackerman, 1998) and work content having a shorter lifecycle (Osman-Gani and Jacobs, 2005), workplace learning has become a widely discussed issue (Eraut, 2004; Lohman, 2005; Jacobs and Park, 2009). Among the different forms of adult workplace learning, informal learning has attracted much recent research attention due to its significance to the development of both individuals and organizations (Jacobs and Park, 2009). Based on the social-cultural theory of learning (Vygotsky, 1978; Lave and Wenger, 1991), informal workplace learning recognizes that, outside of the learning that takes place in structured and externally organized programs, knowledge and skills are also acquired incidentally within the work setting. At present there is no singular definition or unified approach to what informal learning is, largely due to the intersecting interests, contested ideas, and multiple approaches in the field (Manuti et al., 2015). However, a consensus has formed around certain features of informal learning.

First, informal learning often occurs as people carry out their work and acquire the necessary competence to meet current and future work requirements (Jacobs and Park, 2009). Therefore, the needs of organizational development and the needs of individuals who aim to advance their work-related interests and goals serve as key sources of motivation of informal learning (Muhamad and Idris, 2005). Second, informal learning is usually incidental or, in other words, not necessarily conscious. Marsick and Watkins (2001) highlighted incidental learning as a category of informal learning that always takes place outside of a person’s consciousness. Eraut (2000, 2004) differentiated three cognitive processes of informal learning and labeled them instant/reflex, rapid/intuitive, and deliberative/analytic. Therefore, informal learning is usually unplanned and loosely organized, and happens without any clear learning structure or outcome evaluation (Jacobs and Park, 2009). Third, despite being typically incidental and sometimes unconscious and non-intentional, informal learning is always self-initiated and can thus also be strongly intentional, with learning content and form being determined by the learning individuals. Fourth, informal learning can result in individuals and teams refocusing and fundamentally changing their behavior (Garavan et al., 2002; Lohman, 2005). Finally, Marsick and Watkins (2001) highlighted that informal learning activities proceed through an inductive process of reflection and are linked to the learning of others, which points to two main categories of informal learning activities, namely learning through interaction and learning through reflection.

While much of the existing research on informal workplace learning has focused on typical white-collar employees, its role in teaching has also been discussed. Following this research tradition, we conceptualize informal teacher learning (ITL) as the spontaneous and unorganized learning behavior that permeates the daily lives of teachers (Kyndt et al., 2016). In this study, we focus on those intentional ITL activities that are actively organized and initiated by teachers. Such ITL primarily occurs within the school environment but can extend into teachers’ daily lives.

Drawing on Marsick and Watkins (2001) understanding of informal learning, we adopt a distinction between learning through interaction and learning through reflection. Four kinds of interactive ITL have been proposed in the literature, namely learning through (1) media, (2) colleague interaction, (3) stakeholder (e.g., parents and friends) interaction, and (4) student interaction (Kwakman, 2003; Henze et al., 2009). In addition, teachers have reported that they learn reflectively by deliberating on curriculum refinement and instructional improvement (Kolb, 2014; Richter et al., 2011). Teachers have repeatedly attested to transformations in beliefs and improvements in knowledge and skills arising from their engagement in various ITL activities (Grosemans et al., 2015; Kyndt et al., 2016). In sum, this study conceives of ITL activity taking place in five ways, comprising four types of interaction (learning through media, colleague interaction, stakeholder interaction, and student interaction) and individual reflection.

### Teacher Emotions

Emotions are ubiquitous in school and classroom contexts, where learners and teachers come together. In the field of emotions in the classroom, the majority of research has been performed on students (Pekrun and Linnenbrink-Garcia, 2014), with less attention directed to teachers (Frenzel, 2014). Three basic emotions have been acknowledged as the most salient among teachers: enjoyment, anxiety, and anger (Frenzel, 2014; Hagenauer et al., 2015). Enjoyment is one of the most salient positive emotions that teachers experience, either from anticipating a desirable event (anticipatory joy) or from being involved in an activity that in and of itself is experienced as satisfying (activity-related joy; Sutton and Wheatley, 2003; Keller et al., 2014). In teacher education studies, there is ample empirical evidence for the significance of teacher enjoyment for student motivation (Frenzel et al., 2018), student performance (Pekrun et al., 2011), teacher interpersonal relationships (Hagenauer et al., 2015), and teacher well-being (Taxer and Frenzel, 2015). Anxiety refers to an anticipation of future danger, and includes not only cognitive components (concerns, worries, or handling tough situations) but also physiological components (sweating, insomnia, problems with decision making; American Psychiatric Association, 2013). Anxiety has been shown to be negatively associated with teachers exhibiting instructional behavior that supports student learning and enthusiasm (Frenzel et al., 2009), and with low levels of acceptance of errors (Frenzel et al., 2016).

Anger is a negative emotion that can be aroused when there is someone to blame for undesirable events (Kuppens et al., 2003). As the most prominent of all the negative emotions, due to its high frequency and intensity (Becker, 2011), anger is linked with undesirable teaching strategies, including fast-paced instruction and being disrespectful of students (Frenzel et al., 2016), and is negatively linked with teacher well-being (Taxer and Frenzel, 2015).

### Linking Teacher Emotions With ITL

Prior evidence has demonstrated the importance of emotions for teaching behavior (Frenzel et al., 2009, 2016). In the Chinese context, there have been studies related to teacher emotions and curriculum change (Lee and Yin, 2011) and on the connections between teachers’ emotional labor and well-being (Yin et al., 2017, 2018). However, there is a conspicuous lack of empirical findings regarding the effects of teachers’ emotional experiences on their own ITL activities. The deficiency of research into the relationship between emotions and teacher learning has been repeatedly emphasized in related studies. For example, Hoekstra (2007) observed that “research on teacher learning is mostly concerned with teachers” change in cognition’ (p. 116), and Korthagen (2017) called for the integration of emotion into studies of teacher learning. Therefore, empirical studies exploring the associations between emotion and learning among teachers promise to meet a research need.

Emotions are considered inseparable components of the learning process (Pekrun, 2006; Moreno and Mayer, 2007; Plass and Kaplan, 2016), and Mayer (2019) contends that learners’ emotional states should be integrated in the causal chain for explaining learning activity and outcomes. The effects of positive emotions on learning can be illustrated from various angles. First, emotions influence learning performance through their connection with memory (Parrott and Spackman, 2000). If the activity is the object of the emotion, positive emotions can draw on working memory resources that help activity performance. Second, emotions are closely related to learning motivation. Positive emotions (happiness, enjoyment) can promote intrinsic and extrinsic learning motivation among individuals, which is a precursor for learning effort investment (Loderer et al., 2018). Third, emotions can influence information processing (Kuhbandner et al., 2011). Kuhbandner and Pekrun (2013) contended that positive emotions can promote relational and flexible information processing by affecting the storage and retrieval of memory materials. Fourth, it has also been discovered that positive emotions can promote the flexible use of deep learning strategies (Zeidner, 1998; Ahmed et al., 2013; Pekrun and Perry, 2014; Ranellucci et al., 2015). Compared with the positive association between positive emotions and learning, the relationship between negative emotions (such as anxiety and anger) and learning is more complicated. Studies have indicated that negative emotions can undermine intrinsic motivation and learning interaction, but also that they can promote extrinsic learning motivation to avoid possible failure and induce rehearsal-based learning (Loderer et al., 2018). In general, the negative effects of negative emotions on overall learning behavior and outcomes are likely to outweigh the short-term benefits for most learners (Pekrun et al., 2011; Zeidner, 2014). Given the universal features of the appraisal pattern across different learning contexts (Pekrun and Perry, 2014), the association between emotions and learning can be applied to both formal and informal learning (Goetz et al., 2006; Pekrun et al., 2011; Graesser, 2019).

### Considering Teaching Experience for Teacher Emotions and ITL

We considered our theoretical framework with reference to a teacher career cycle approach highlighting the importance of teaching experience, which has been explored by a number of educational researchers (Huberman, 1989, 1993; Hargreaves, 2005; Day et al., 2009) with respect to several outcomes: teacher self-efficacy (Tschannen-Moran and Hoy, 2007), teacher commitment (Klassen and Chiu, 2011), teacher learning (Richter et al., 2011), and teacher burnout (Antoniou et al., 2006).

Some studies have shown that teachers’ emotional experiences can be related to teaching experience. Younger teachers have been reported to experience higher levels of professional burnout (Antoniou et al., 2006) and anxiety (Sutton and Wheatley, 2003; Chang, 2009). Regarding teacher learning, Cameron et al. (2013) reported that beginning teachers showed a greater need for professional development and a higher motivation for learning, which together lead to more frequent learning behavior. Richter et al. (2011) found that experienced teachers use more professional literature but less teacher collaboration compared with novice teachers. As such, there is initial empirical evidence for teachers’ emotions and learning behavior varying with teaching experience, which is why we sought to also explore those links in the present study.

### The Chinese Context

Our study was conducted in China, which implements a system of 9 years of compulsory education, comprising 6 years of primary education and 3 years of junior secondary education. In China, under the influence of its collectivist-cooperative culture, teachers value relationship-building and self-reflection for improvement (Qi et al., 2007; Law, 2012). The results of several empirical studies indicate that Chinese teachers are happy to work with students and enjoy the simplicity and safety of campus life, but are dissatisfied with a lack of opportunities for continuous professional development and high levels of work stress (Song, 2007; Sun et al., 2008). Overall, Chinese teachers tend to enjoy a higher status than most other occupations, but there are reports of teachers feeling they are not objectively and appropriately valued by society as a whole (Liu and Onwuegbuzie, 2014).

## Hypotheses

In response to a notable lack of research addressing the association between teacher emotions and ITL, this study of primary school teachers in China examined the levels of and relationships between those variables, while also considering teaching experience. Based on previous research findings, we proposed the following two hypotheses.

**Hypothesis 1**: Based on compelling theoretical reasoning and prior evidence that emotions are closely linked with information processing, memory, self-regulated learning, and motivation, we expected a positive relationship between the positive emotion of enjoyment and ITL activities, and negative relationships between the negative emotions of anxiety and anger and ITL activities.
**Hypothesis 2**: We posited differences in levels of key study variables with teaching experience. Based on teaching career cycle reasoning (Huberman, 1989; Day et al., 2007) and prior findings on teacher emotions (Sutton and Wheatley, 2003; Chang, 2009; Day and Gu, 2014), we predicted teacher enjoyment to be positively related to years of experience, and anxiety and anger to be negatively related to years of experience. Drawing from the findings on teacher learning (Cameron et al., 2013; Kyndt et al., 2016), we also expected that ITL activities would be negatively linked with years of experience.

## Research Method

To address the hypotheses posited in this study, we adopted an exploratory correlational design to investigate the links between teacher emotions and ITL activities.

### Participants and Procedure

Our sample was made up of 2,880 primary school teachers (85.49% female) recruited from Chongqing in the southwest of China. This study was approved by the Human Research Ethics Committee of the University of Hong Kong. All participating teachers gave their consent before participating in the study, and were recruited on a voluntary basis. In China, most teachers have a personal account on WeChat (an online instant messaging platform) and join various teacher groups organized by the municipal educational commission. These groups are specific to different levels of schooling (early childhood, primary, elementary, tertiary). The link to the questionnaire was directly sent to online groups for elementary teacher. School type was part of the demographic information provided by teachers, and only primary teacher data were kept for data analysis in the present study. The mean age of the respondents was 36.32 years (*SD* = 8.90) and the mean years of experience was 15.00 (*SD* = 10.18). Just over half (53.65%) of the participants were teaching at city schools and the others were teaching in rural districts. In terms of educational attainment, 3.13% of the teachers held a Master’s degree or above, 74.86% held a Bachelor’s degree, and 21.01% held an associate degree.

### Measures

#### Teacher Emotions

Teachers’ self-reported experiences of enjoyment, anger, and anxiety during teaching were measured by the Teacher Emotions Scale (TES) developed by Frenzel et al. (2016). The TES has 12 items covering the three discrete emotions of enjoyment, anxiety and anger. The Chinese translation of the scale was coordinated by the second author. The items were firstly translated from English into Chinese. To validate the item translation, the Chinese version was then translated back into English for checking the conceptual equivalence with the original English items. The questionnaire was then sent to two Chinese scholars in teacher education to ensure that the items were naturally and practically presented. Examples of items on the TES include “I generally enjoy teaching” for enjoyment (α = 0.94), “Preparing to teach often causes me to worry” for anxiety (α = 0.88), and “I often feel annoyed while teaching” for anger (α = 0.90). Responses were marked on a 4-point Likert scale ranging from 1 (*strongly disagree*) to 4 (*strongly agree*). The full set of items in the TES-Chinese is presented in Table A2.

#### ITL

Based on open-ended interviews with 10 teachers from various backgrounds working at three different schools, and with reference to the relevant literature (Kwakman, 2003; Kyndt et al., 2016; Louws et al., 2017), the authors developed and revised an ITL scale made up of 18 items designed to measure the frequency with which the participating teachers had carried out different ITL activities over the previous 6 months. The resulting ITL scale included five dimensions: learning through media, colleague interaction, stakeholder interaction, student interaction, and reflection (the list of items is given in Tables A1, A2 in English and Chinese, respectively). Responses were marked on a 5-point Likert scale ranging from 1 (*never*) to 5 (*always*). Cronbach’s alpha ranged from 0.79 to 0.95 for the five dimensions in the present study, indicating acceptable internal consistency.

### Analyses

A multivariate normality test revealed that our data were not normally distributed. Considering that the latent variables in this study were derived from multi-item indicators, they can be considered continuous variables; therefore we used the MLR (robust maximum likelihood estimator) with robust standard errors (Finney and Christine, 2006) for parameter estimation. To test the construct validity of the variables under study, we randomly split our data into two datasets. With one dataset, exploratory factor analyses (EFA) with the MLR extraction method and oblique rotation method were applied to explore the internal construct validity of the TES and ITL, respectively. Three factors of TES and five factors of ITL emerged. All items showed high factor loadings with their corresponding factors (λ ≥ 0.33; see Table A1 for all the items with factor loadings). Next, two separate CFAs were conducted with the other half of the data set to further confirm the 3- or 5-factor structure of the TES and ITL, respectively. The model fit was assessed using the chi-square value, CFI, TLI, RMSEA, and SRMR. We deemed model fit acceptable when CFI and TLI were no less than 0.90, and RMSEA and SRMR were below 0.08 (Schreiber et al., 2006). Both models revealed a good model fit (see Table 1), and the measurement weights of all items with their corresponding measures were sufficiently high (≥0.65; see Table A1). The fit indices of the measurement model revealed good model fit (RMSEA = 0.05, CFI = 0.96, TLI = 0.95, SRMR = 0.04).

**TABLE 1:** CFA results for confirming the internal factor structures for the TES and ITL.

**Scale**	**CFI**	**TLI**	**RMSEA**	**SRMR**
TES	0.98	0.97	0.05	0.03
ITL	0.98	0.98	0.04	0.03

We used linear regression to investigate the links between teaching experience with teacher emotions and ITL. The level of significance was specified as 0.05. To test the associations between teacher emotions and ITL, structural equation modeling (SEM) was carried out with the three emotions as criteria, and the five ITL activities as outcomes. The model showed a good fit (RMSEA = 0.05, CFI = 0.96, TLI = 0.95, SRMR = 0.04).

## Results

### Descriptive Statistics and Correlations Between Eight Variables

The means and standard deviations of each variable and the correlations between them are shown in Table 2. Teachers most strongly endorsed the items pertaining to enjoyment (*M* = 3.24, *SD* = 0.58) and least endorsed the items for anger (*M* = 2.12, *SD* = 0.74). Of the ITL activities, teachers scored highest on learning through colleague interaction (*M* = 4.43, *SD* = 0.75) and lowest on learning through student interaction (*M* = 3.90, *SD* = 0.94). The size of the correlations between the variables ranged from −0.45 to 0.79.

**TABLE 2:** Descriptive statistics, Cronbach’s alpha, and correlations between the eight variables (*N* = 2880).

**Variables**	**Mean**	**SD**	**1**	**2**	**3**	**4**	**5**	**6**	**7**	**8**
1. TES-enjoyment	3.24	0.58	1							
2. TES-anxiety	2.19	0.74	−0.39***	1						
3. TES-anger	2.12	0.74	−0.45***	0.79***	1					
4. ITL-media	3.91	0.83	0.49***	−0.24***	−0.27***	1				
5. ITL-colleague interaction	4.43	0.75	0.41***	−0.24***	−0.24***	0.68***	1			
6. ITL-stakeholder interaction	3.97	0.89	0.49***	−0.21***	−0.24***	0.75***	0.70***	1		
7. ITL-student interaction	3.90	0.94	0.47***	−0.19***	−0.25***	0.66***	0.55***	0.73***	1	
8. ITL-reflection	4.37	0.71	0.48***	−0.26***	−0.27***	0.67***	0.69***	0.67***	0.65***	1
Cronbach’s α			0.94	0.88	0.90	0.85	0.95	0.79	0.93	0.92

*p < 0.05, **p < 0.01, ***p < 0.001.

### Linking Teacher Emotions and ITL Activities

**FIGURE 1:**
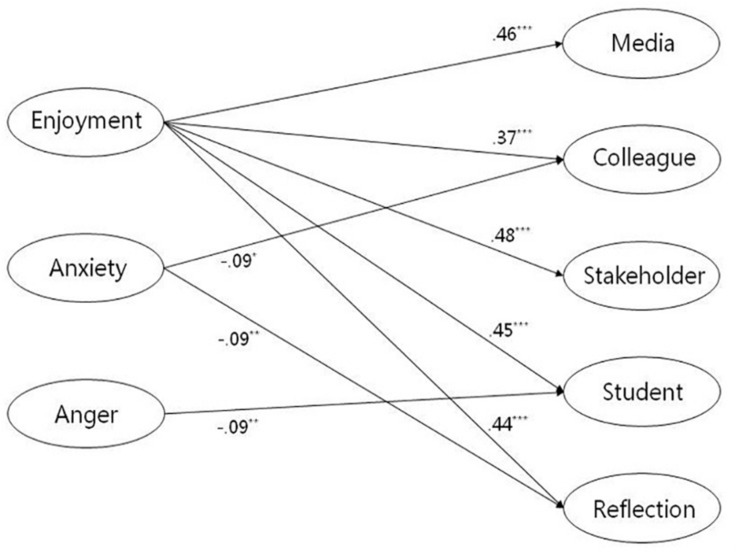
Structural equation model for the relationship between teacher emotions and ITL activities. Manifest indicator intercepts and errors as well as non-significant regression paths are not shown. Standardized regression coefficients are reported. *p < 0.05, **p < 0.01, ***p < 0.001.

Regarding the relationship between teacher emotions and ITL activities (see Figure 1), enjoyment was significantly positively related to learning through stakeholder interaction (β = 0.48, *p* < 0.001), media (β = 0.46, *p* < 0.001), student interaction (β = 0.45, *p* < 0.001), individual reflection (β = 0.44, *p* < 0.001), and colleague interaction (β = 0.37, *p* < 0.001). Anxiety was negatively associated with learning through colleague interaction (β = −0.09, *p* < 0.05) and individual reflection (β = −0.09, *p* < 0.01). Anxiety was not significantly linked with the other three ITL activities (β_*media*_ = −0.02, *p* = 0.60; β_*stakeholder*_ = −0.02, *p* = 0.53; β_*student*_ = 0.06, *p* = 0.07). Anger was negatively related to student interaction (β = −0.09, *p* < 0.01) but not to the other four ITL activities (β_*media*_ = −0.05, *p* = 0.14; β_*colleague*_ = −0.01, *p* = 0.87; β_*stakeholder*_ = −0.01, *p* = 0.84; β_*reflection*_ = −0.01, *p* = 0.84). These results partially supported Hypothesis 1.

### Links With Teaching Experience

The second hypothesis, which predicted that teacher emotions and ITL would be significantly related to teaching experience, was partially supported by the data, with only linear trends being found. Specifically, as shown in Table 3, we found that anxiety (β = −0.11, *p* < 0.001) and anger (β = −0.10, *p* < 0.001) significantly linearly decreased with years of experience. No correlation was found between teaching experience and enjoyment (β = 0.01, *p* = 0.48).

**TABLE 3:** Links between teaching experience and three teacher emotions.

	**Teacher emotions**		
	**Enjoyment**	**Anxiety**	**Anger**
Years of teaching	0.013	−0.110***	−0.102***
	(0.703)	(−5.883)	(−5.337)
*R*^2^	0.000	0.012	0.010

Standardized beta coefficients; t statistics in parentheses. *p < 0.05, **p < 0.01, ***p < 0.001.

Regarding the five ITL activities, reflection (β = 0.11, *p* < 0.001), student interaction (β = 0.08, *p* < 0.001), learning through media (β = 0.078, *p* < 0.001), and colleague interaction (β = 0.06, *p* < 0.01) were positively related to teaching experience, whereas no correlation was found between teaching experience and learning through stakeholder interaction (β = −0.02, *p* = 0.24) (see Table 4).

**TABLE 4:** Links between teaching experience and the five ITL activities.

	**ITL activities**				
	**Media**	**Colleague**	**Stakeholder**	**Student**	**Reflection**
Years of teaching	0.078***	0.063**	−0.022	0.078***	0.111***
	(4.194)	(3.371)	(−1.186)	(4.211)	(6.001)
*R*^2^	0.006	0.004	0.001	0.006	0.012

Standardized beta coefficients; t statistics in parentheses. *p < 0.05, **p < 0.01, ***p < 0.001.

## Discussion

This study investigated the associations between teacher emotions and ITL, and examined the relationships between teaching experience with teacher emotions and various ITL activities. The results yielded four major findings. First, teacher enjoyment was positively related to all ITL activities. Second, anxiety was negatively associated with learning through colleague interaction and individual reflection. Third, anger was negatively related to learning through student interaction. Fourth, teaching experience was a significant correlate and had monotonic relationships with teacher anger and anxiety, and some of the ITL activities.

### Complex Relationships Between Teacher Emotions and ITL

Our first hypothesis concerned the relationship between teacher emotions and ITL activities. The results of this study generally confirm the bivariate relationships between emotions and learning highlighted in previous studies (Ahmed et al., 2013; Kuhbandner and Pekrun, 2013; Pekrun and Perry, 2014) while expanding this to the context of teachers, gauging the function of enjoyment, anxiety, and anger on each of five ITL activities.

The findings of this study indicate that teachers who feel joyful about teaching are keen to pursue updated educational information and to discuss teaching issues with students, colleagues, and stakeholders. This may be reflective of the close links between enjoyment and learning motivations (Loderer et al., 2018), flexible information access and information processing (Kuhbandner and Pekrun, 2013). Enjoyment was also found to have a significant link with teachers’ propensities to reflect on their own teaching, which might be explained by the associations between positive emotions and deep learning strategies (Ahmed et al., 2013; Pekrun and Perry, 2014).

Concerning the relationship between anxiety and ITL, we found a negative association with colleague interaction and reflection. It has been reported that teacher collaboration may cause work intensification (Johnson, 2003) and anxiety (Musanti and Pence, 2010). If anxiety stemming from collaboration is negatively perceived by teachers, then these anxious teachers are likely to avoid intensive colleague interactions. Furthermore, we found that anxiety was negatively related to teacher reflection. This may relate to the cognitive interference caused by anxiety (Zeidner and Matthews, 2005), which is negatively related to encoding, information storing, and processing (Hembree, 1998; Zeidner, 2014), and thus may deter self-reflection.

Regarding the relationship between anger and ITL activity, we found that anger among teachers was independent of engagement in most ITL activities, except for a negative relationship with learning through student interaction. Studies have indicated that student non-conformance with classroom rules (Hargreaves, 2000) and general student misbehavior (Chang, 2009) are the most salient reasons for anger among teachers (Frenzel, 2014). It would not be surprising that if there are strong tensions in teacher–student relationships that bring about teacher anger, then this will constrain teacher–student interactions about teaching and thus limit opportunities for teachers to learn through such interaction.

### Monotonic Relationships Between Years of Experience With Teacher Emotions and Various ITL Activities

Our second hypothesis predicted links in the levels of various study variables with years of experience, and was largely supported by the study results, with the exception of enjoyment. The data showed that teacher enjoyment was independent of years of experience, whereas anxiety and anger were negatively related to years of experience. In general, the enjoyment of teachers with various years of experience was found to remain at a relatively high level, indicating that the study participants generally enjoyed their teaching across all ages. However, this does not mean that they were not subject to negative emotions. We found relatively higher levels of negative emotions (*M*_*anxiety*_ = 2.19 and *M*_*anger*_ = 2.12) than comparable studies conducted in Western contexts (*M*_*anxiety*_ = 1.44 and *M*_*anger*_ = 1.88 in Frenzel et al., 2016). Furthermore, novice teachers were found to be more vulnerable to negative emotions than their counterparts with many years of experience. The higher levels of anxiety and anger among novice teachers may arise from their lack of familiarity with the subject matter, concerns about losing control of classroom management (Bibby, 2002; Intrator, 2006), and their sensitiveness to negative feedback from students (Stough and Emmer, 1998). It is worth noting that the effect sizes for these links with teaching experiences were generally small.

The sizes of the links between years of experience and ITL activities were also small, and they differed across the five ITL activities. Similar to Richter et al. (2011), this study confirms that senior teachers access more professional literature than novice teachers do. Furthermore, we found that senior teachers engaged in more student interaction, colleague interaction, and reflection than novice teachers. This may be because novice teachers often struggle to manage teaching challenges, student problems and overwhelming emotions (Intrator, 2006; Day et al., 2007). These unsolved problems coupled with negative emotions may limit their willingness and openness to discuss teaching with students or to contemplate engaging in instructional improvement together with colleagues.

## Limitations and Future Directions

A few limitations of this study need to be considered when interpreting the findings and devising future research directions. First, this study relied on self-reported measures to assess teacher emotions. Future studies could integrate physiological measures, external observer ratings, or student reports to triangulate self-reported emotions. Second, the teachers in this study were all teaching at primary schools. The relationships between the studied variables may vary across different school settings (e.g., kindergarten or secondary school) or educational contexts. Third, ITL is subject to many other factors, such as school environment, personal goal orientation, and learning motivation (Kyndt et al., 2016). Future studies should include additional factors to gain a more comprehensive picture of ITL correlates, which could contribute to the establishment of a theoretical model of ITL. Last, as Pekrun et al. (2014) and Frenzel (2014) noted, emotions, their antecedents, and their outcomes are linked by reciprocal causation. To gain a better insight into the temporal causal dynamics that underlie the correlative links between teacher emotions and ITL activities reported in the present study, future research should adopt longitudinal study designs or experimental approaches.

## Practical Implications

The findings of this study yield two key practical implications for teacher trainers and administrators. The significant role that teacher emotions seem to play for ITL engagement indicates the importance of supporting teachers in enhancing their positive emotions. Based on control-value theory, Pekrun and Perry (2014) advanced three approaches that can be adopted in school settings. The first is an appraisal-oriented approach that focuses on improving positive emotions by supporting individuals to focus on their successes and thus positively evaluate their abilities (Frenzel and Stephens, 2013). The second is a situation-oriented approach looking at various strategies that schools can use to improve teachers’ enjoyment in their accomplishments. Some strategies have been explored for constructing emotion-friendly environments (e.g., interest-enhancing strategies and relaxation techniques; see Sansone et al., 1992). The third approach advanced by Pekrun and Perry (2014) is competence-oriented, building on the notion that positive emotions arise from improvements in competence. For example, improved classroom management skills or student communication skills can decrease negative emotions and increase positive emotions (Sutton, 2007; Jacob et al., 2017). Therefore, sustainably facilitating teacher professional development can contribute to teachers experiencing more positive emotions.

Second, given the negative association between anxiety and colleague interaction and reflection found in this study, teacher trainers and principals should take anxiety into consideration when designing any programs targeting teacher collaboration and reflection, especially for beginning teachers. Regarding teacher reflection, scholars have mainly focused on the cognitive aspects (e.g., Schön, 1987; Mezirow, 2000). This study highlighted the significant relationship between emotion and reflection, which suggests benefits to integrating emotional components to the design of reflection improvement programs.

## Conclusion

The present study contributes to the current body of knowledge on ITL and teacher emotions by examining the relationships between three teacher emotions and five ITL activities, while also taking account of the role of teaching experience. Teacher enjoyment was found to be positively associated with all five ITL activities. Teacher anxiety was negatively related to learning through colleague interaction and self-reflection on teaching, and anger was negatively related to student interaction. The results further revealed a monotonic relationship of teaching experience with five kinds of ITL activities and three emotions. Specifically, it was found that that experienced teachers engaged in more reflection, student and colleague interactions, and on- and off-line reading than beginning teachers. Teacher anxiety and anger were found to be negatively associated with years of experience, and enjoyment was steady over the career course.

## Data Availability Statement

The datasets generated for this study are available on request to the corresponding author.

## Ethics Statement

The studies involving human participants were reviewed and approved by Human Research Ethics Committee, The University of Hong Kong. Written informed consent for participation was not required for this study in accordance with the national legislation and the institutional requirements.

## Author Contributions

XH, JL, and AF contributed conception and design of the study. XH organized the database, performed the statistical analysis, and wrote the first draft of the manuscript. All authors contributed to manuscript revision, read and approved the submitted version.

## Conflict of Interest

The authors declare that the research was conducted in the absence of any commercial or financial relationships that could be construed as a potential conflict of interest.

## Figures and Tables

**TABLE A1:** Standardised factor loadings for the items in each construct.

**Construct and items**	**Factor loading (λ)**	**Measurement weight**
**Learning through media**		
I have browsed educational materials (e.g., educational papers or reports) on the internet.	0.79	0.75
I have browsed teaching materials (e.g., lesson plans, videos of public lessons) on the internet.	0.61	0.75
I have read education-related posts on social-media platforms, such as Facebook, Quora etc.	0.73	0.70
I have read hard-copy educational materials (e.g., teaching reference books, journals).	0.33	0.65
I have sought out materials (e.g., newspapers, movies) that could provoke me to think about education.	0.50	0.79
**Learning through colleague interaction**		
I have communicated with my colleagues about curricula and teaching.	0.90	0.92
I have communicated with my colleagues about student learning.	0.94	0.95
I have communicated with my colleagues about teaching problems.	0.83	0.91
**Learning through stakeholder interaction**		
I have communicated with parents about educational issues.	76	0.80
I have communicated with my family or friends about educational issues.	0.88	0.82
I have discussed educational issues in online communities.	0.46	0.66
**Learning through student interaction**		
I have discussed teaching materials or lesson content with my students outside of regular class hours.	0.87	0.91
I have discussed teaching methods with my students.	0.96	0.94
I have discussed my teaching effectiveness with my students.	0.86	0.91
I have talked with students about any topics they are interested in.	0.44	0.74
**Learning through individual reflection**		
When my teaching has not met my expectations, I have considered possible reasons why.	0.77	0.86
I have thought about or learned from my teaching successes.	0.91	0.93
I have thought about how to continue to improve my teaching.	0.92	0.91
**Enjoyment**		
I generally enjoy teaching.	0.86	0.87
I generally have so much fun teaching that I gladly prepare and teach my lessons.	0.92	0.91
I generally teach with enthusiasm.	0.90	0.90
I often have reason to be happy while I teach.	0.87	0.90
**Anxiety**		
I generally feel tense and nervous while teaching.	0.64	0.77
I am often worried that my teaching isn’t going well.	0.81	0.73
Preparing to teach often causes me to worry.	0.92	0.84
I feel uneasy when I think about teaching.	0.78	0.87
**Anger**		
I often have reason to be angry while I teach.	0.94	0.86
I often feel annoyed while teaching.	0.85	0.90
Sometimes I get really mad while I teach.	0.81	0.79
Teaching generally frustrates me.	0.51	0.78

*The factor loadings emerged from two separate EFAs using the principal components analysis extraction method and Kaiser normalisation oblimin rotation method. The measurement weights were generated from two separate CFAs confirming the 3- or 5-factor structure of the TES and ITL.*

**TABLE A2:** Chinese version of all survey items.